# Continued Atlantic overturning circulation even under climate extremes

**DOI:** 10.1038/s41586-024-08544-0

**Published:** 2025-02-26

**Authors:** J. A. Baker, M. J. Bell, L. C. Jackson, G. K. Vallis, A. J. Watson, R. A. Wood

**Affiliations:** 1 Met Office, Exeter, UK; 2 University of Exeter, Exeter, UK

**Keywords:** Physical oceanography, Projection and prediction, Physical oceanography, Climate and Earth system modelling

## Abstract

The Atlantic Meridional Overturning Circulation (AMOC), vital for northwards heat transport in the Atlantic Ocean, is projected to weaken owing to global warming^1^, with significant global climate impacts^2^. However, the extent of AMOC weakening is uncertain with wide variation across climate models^1,3,4^ and some statistical indicators suggesting an imminent collapse^5^. Here we show that the AMOC is resilient to extreme greenhouse gas and North Atlantic freshwater forcings across 34 climate models. Upwelling in the Southern Ocean, driven by persistent Southern Ocean winds, sustains a weakened AMOC in all cases, preventing its complete collapse. As Southern Ocean upwelling must be balanced by downwelling in the Atlantic or Pacific, the AMOC can only collapse if a compensating Pacific Meridional Overturning Circulation (PMOC) develops. Remarkably, a PMOC does emerge in almost all models, but it is too weak to balance all of the Southern Ocean upwelling, suggesting that an AMOC collapse is unlikely this century. Our findings reveal AMOC-stabilizing mechanisms with implications for past and future AMOC changes, and hence for ecosystems and ocean biogeochemistry. They suggest that better understanding and estimates of the Southern Ocean and Indo-Pacific circulations are urgently needed to accurately predict future AMOC change.

## Abstract (web summary)

Climate models suggest that the Atlantic Meridional Overturning Circulation is unlikely to collapse this century, owing to stabilization from wind-driven upwelling in the Southern Ocean.

## Main

The future state of the Atlantic Meridional Overturning Circulation (AMOC) is critical for global and regional climate change^2^ through its role in heat transport^6^ and carbon uptake^7^. The AMOC is predicted to weaken during the twenty-first century^1^, driven by increased greenhouse gas (GHG) concentrations and freshwater input to the North Atlantic owing to precipitation changes and Greenland Ice Sheet melt^8,9^. However, climate model projections from the Coupled Model Intercomparison Project Phase 6 (CMIP6) vary widely^1,3,4^, so its future evolution is uncertain^10^. Furthermore, there is a risk that the AMOC could collapse, causing abrupt changes in climate^11,12^. Several statistical indicators suggest that it is approaching a tipping point^5,13,14^, indicating that climate models may be overstable^15^. As AMOC changes will impact many aspects of the climate^2^, accurately predicting future AMOC change is vital for planning adaptation and mitigation strategies.

Here we examine the AMOC’s response to extreme GHG and North Atlantic freshwater forcings in CMIP6 climate models. Although simpler models^12,16^ and a few global climate models suggest that the AMOC could collapse (that is, weaken to zero or reverse) under such forcings^14,17,18^, it does not collapse in the model experiments considered here. Instead, the AMOC levels off at a weaker strength that varies widely across models^2,19^.

To understand how the AMOC is sustained under extreme climate forcing, and what causes differences in its weakened state across models and forcings, we quantify the AMOC’s upwelling pathways^20^, which return deep North Atlantic waters to the surface either through Southern Ocean (SO) wind-driven upwelling^21,22^ or diffusion in the Atlantic and Indo-Pacific oceans^23^. The SO wind-driven upwelling rate depends on the SO westerly wind strength that drives SO upwelling and on the opposing SO mesoscale eddy response, referred to as eddy compensation^24,25^. Our approach, which focuses on AMOC upwelling pathways rather than the mean state of the North Atlantic^4,26^, offers fresh perspectives on the AMOC response. Although the AMOC’s Indo-Pacific upwelling pathway largely controls intermodel variations in AMOC decline in realistic scenarios^3^, the impact of extreme forcing on upwelling pathway changes and thereby potentially AMOC resilience has not been explored. Here we assess how ocean circulation changes remote from the Atlantic affect the AMOC and its upwelling pathways in climate models. Our analysis reveals that SO wind-driven upwelling maintains a weakened AMOC under extreme forcing, overcoming the destabilizing effects of a developing Pacific Meridional Overturning Circulation (PMOC), with implications for understanding both past and future risks of AMOC collapse. We define the PMOC as a clockwise overturning cell in the Indo-Pacific Ocean (Methods), characterized by sinking and densification occurring in the subtropical North Pacific or more southerly latitudes, rather than the more commonly referenced northern latitudes^27–29^.

## Experiments and AMOC upwelling pathways

We examine the AMOC in 34 CMIP6 climate models from the pre-industrial control simulation and their response under two extreme-forcing scenarios (Methods): an abrupt quadrupling of atmospheric carbon dioxide (‘4xCO2’) and a 0.3-Sv North Atlantic freshwater forcing (‘u03_hos’^19^). Only seven of the models are available for u03_hos (Extended Data Table 1), herein named the u03_hos model subset.

**Fig. 1:**
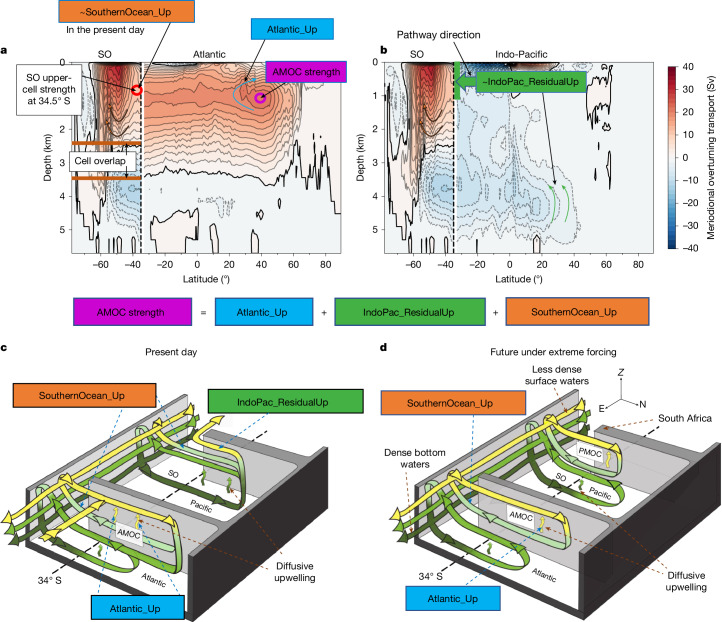
Schematic and analysis method for AMOC upwelling pathways. **a**,**b**, Meridional overturning streamfunction in sverdrups (Sv (10^6^ m^3^ s^−1^), with 2-Sv contour intervals) from the CMCC-ESM2 pre-industrial control simulation, highlighting the methodology for separating the AMOC’s upwelling pathways. Streamfunctions are shown for the Atlantic (**a**) and Indo-Pacific (**b**) oceans north of 34.5° S (indicated by vertical dashed lines) and globally within the Southern Ocean (SO). The 0-Sv streamline is marked by a solid black line and the vertical green line at 34.5° S in **b** denotes the net volume transport from the Indo-Pacific to the Southern Ocean over the highlighted depth. The coloured circles highlight the meridional overturning circulation strength at these locations. The term IndoPac_ResidualUp is calculated as a residual by rearranging the equation shown below the panels. **c**,**d**, Schematic of the AMOC’s upwelling pathways in the present day (**c**) and under scenarios of extreme GHG or North Atlantic freshwater forcing (**d**). Transport pathways are sketched, with increasing water mass density illustrated by a colour gradient from yellow to dark green.

We apply the method of ref. ^3^ to calculate the AMOC’s upwelling pathways (Fig. 1a,b and Methods), which represent the time-mean area-integrated volume transports returning deep waters from the AMOC’s southwards branch to its shallower northwards branch (Fig. 1c). We use the Atlantic, Indo-Pacific and global overturning streamfunctions to quantify these volume transports (Methods). We define the AMOC strength as the maximum strength of the Atlantic mid-depth overturning cell (purple circle in Fig. 1a). The AMOC’s upwelling pathways are defined by the regions where they upwell before rejoining the AMOC’s northwards branch—the Atlantic Ocean (‘Atlantic_Up’), the Indo-Pacific Ocean (‘IndoPac_ResidualUp’) and the Southern Ocean (‘SouthernOcean_Up’). AMOC deep waters that upwell in the Indo-Pacific Ocean and subsequently upwell in the SO are accounted for by SouthernOcean_Up, not IndoPac_ResidualUp (Methods). Together, the three upwelling pathways are equal to the AMOC strength (Fig. 1), ensuring that volume is conserved in the ocean^30^. In the control simulation, SouthernOcean_Up is similar to the SO upper cell strength at 34.5° S (red circle in Fig. 1a), with adjustments for a localized South Atlantic circulation (Methods and Extended Data Fig. 10). In the forcing experiments, where an emergent PMOC causes SO upwelling to balance sinking in both the Atlantic and Indo-Pacific, SouthernOcean_Up approximately equals the AMOC strength at 34.5° S, which may be much weaker than the SO upper cell strength at this latitude (compare Fig. 1c,d).

## Changes in the overturning circulation

**Fig. 2:**
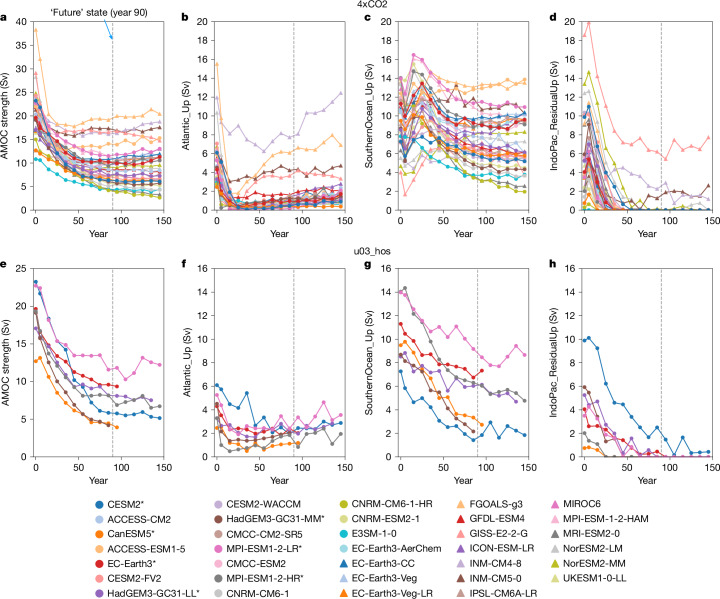
SO upwelling sustains future AMOC strength. **a**–**h** Decadal-mean evolution of AMOC strength and upwelling pathways under extreme-forcing scenarios: abrupt quadrupling of CO_2_ (4xCO2; **a**–**d**) and North Atlantic freshwater hosing (u03_hos; **e**–**h**). Variables plotted are AMOC strength (**a**,**e**), and Atlantic (**b**,**f**), SO (**c**,**g**) and Indo-Pacific residual (**d**,**h**) upwelling pathways of the AMOC. The control simulation averaged over the first 50 years is plotted at year 0, with magnitudes under the forcing scenarios calculated in 10-year intervals. Models used in both the 4xCO2 and u03_hos scenarios are labelled with an asterisk.

**Extended Data Fig. 1:**
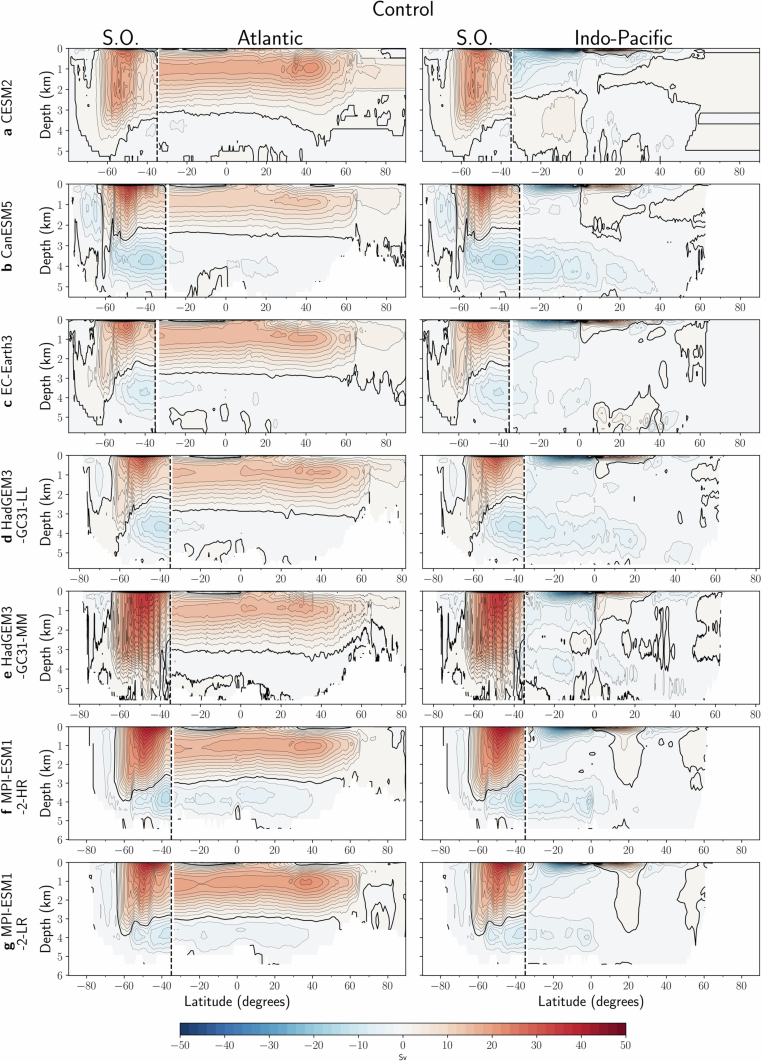
Overturning circulation in the pre-industrial control simulation. Overturning streamfunction (Sverdrups (Sv); 2 Sv contour interval) in depth space averaged over the initial 50 years of the pre-industrial control experiment in the u03_hos subset of models, for the (left) Atlantic, and (right) Indo-Pacific Ocean north of 34.5°S (indicated by vertical dashed lines) and globally within the Southern Ocean (“S.O.”). The zero-streamline contour is defined by the solid black line.

In the pre-industrial control, all models have a clockwise, mid-depth overturning cell (that is, the AMOC) in the Atlantic, an anticlockwise cell in the Indo-Pacific and a clockwise wind-driven upper cell in the SO (Fig. 1a–c and Extended Data Fig. 1). However, models vary widely in AMOC strength and their upwelling pathways (year 0 in Fig. 2a–d).

Under GHG and freshwater forcings, all models show AMOC weakening with a wide spread (Fig. 2a,e), levelling off within 90 years (the ‘future’ state herein). The AMOC weakens by 20–81% (mean of 54%) 90 years after 4xCO2 forcing. Considering only the u03_hos model subset, the weakening ranges from 50–80% in both 4xCO2 (mean of 56%) and u03_hos (mean of 61%). Thus, the future AMOC strength differs across the models, leading to radically different climate impacts^2^.

**Fig. 3:**
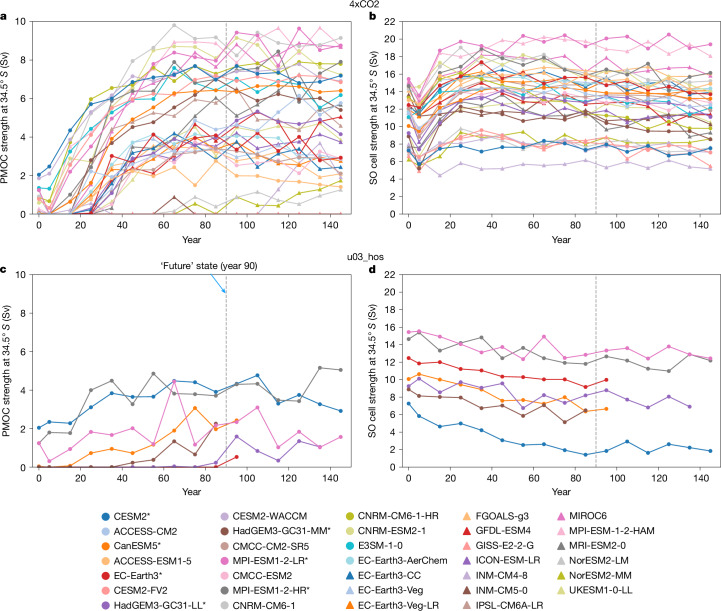
PMOC emerges and SO overturning circulation changes under extreme forcing. **a**–**d**, Decadal-mean evolution in abrupt quadrupling of CO_2_ (4xCO2; **a**,**b**) and North Atlantic freshwater hosing (u03_hos; **c**,**d**,) forcing scenarios. Variables plotted are PMOC strength at 34.5° S (**a**,**c**) and SO upper cell strength at 34.5° S (**b**,**d**). Models used in both the 4xCO2 and u03_hos scenarios are labelled with an asterisk.

**Extended Data Fig. 2:**
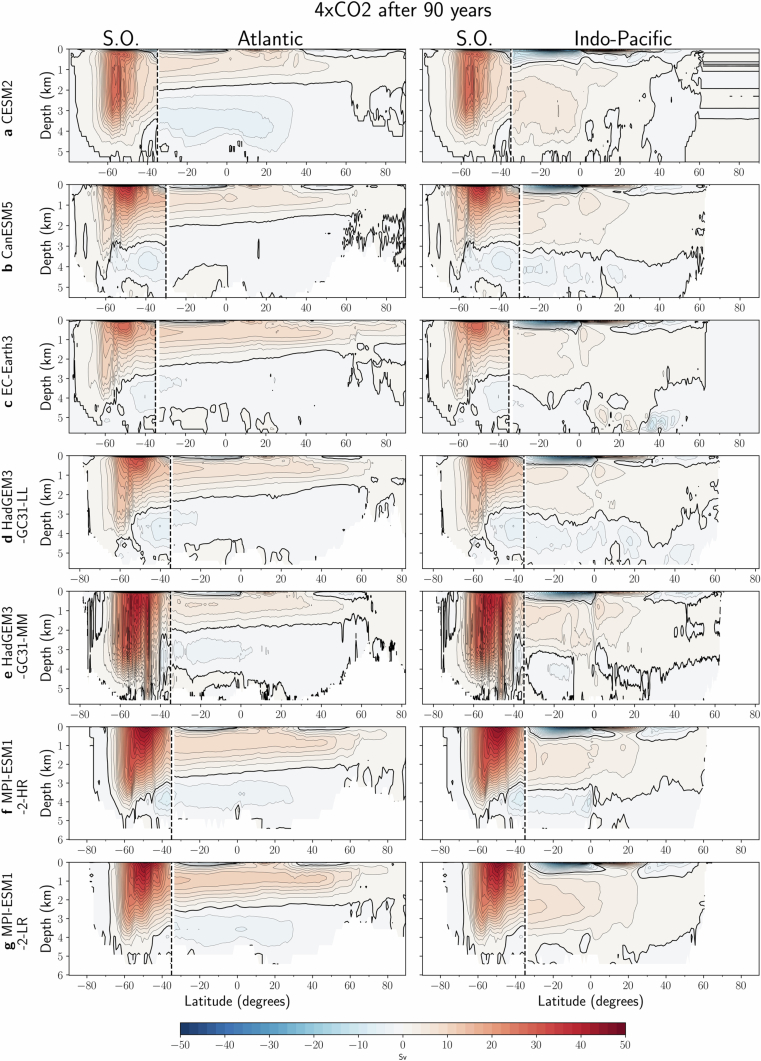
Overturning circulation in the future state of the 4xCO2 experiment. Overturning streamfunction (Sverdrups (Sv); 2 Sv contour interval) in depth space averaged over the 20-year period centred on year 90 of the 4xCO2 experiment in the u03_hos subset of models, for the (left) Atlantic, and (right) Indo-Pacific Ocean north of 34.5°S (indicated by vertical dashed lines) and globally within the Southern Ocean (“S.O.”). The zero-streamline contour is defined by the solid black line.

**Extended Data Fig. 3:**
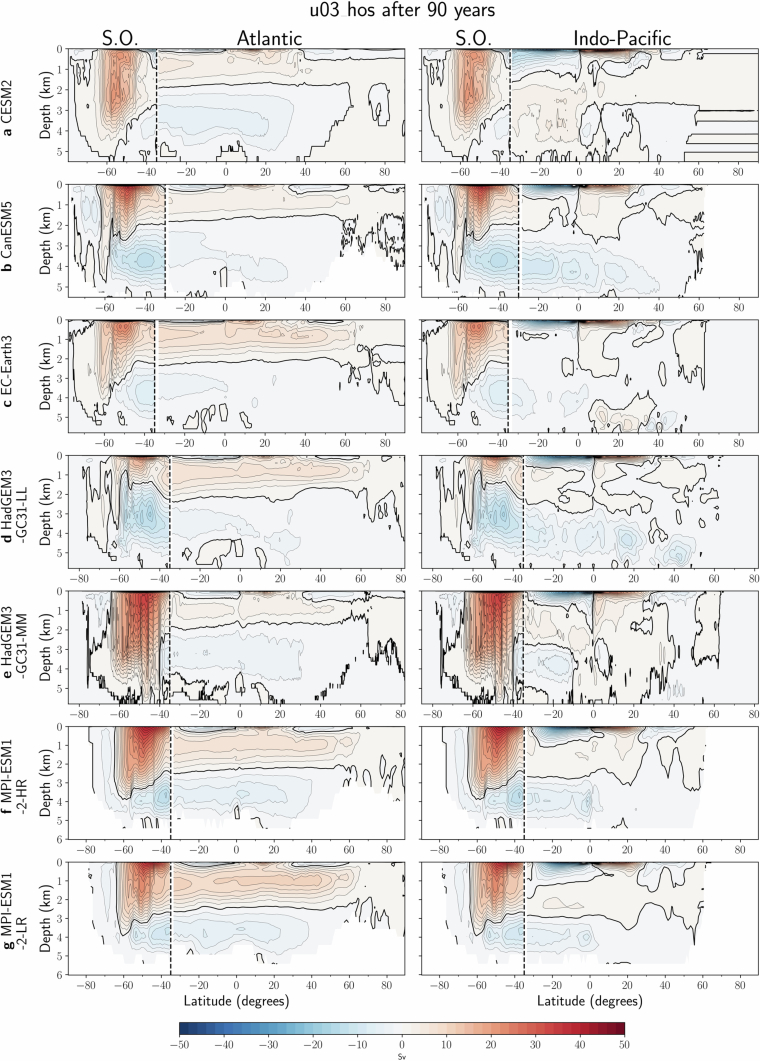
Overturning circulation in the future state of the North Atlantic freshwater forcing experiment. Overturning streamfunction (Sverdrups (Sv); 2 Sv contour interval) in depth space averaged over the 20-year period centred on year 90 of the u03_hos experiment in the models available, for the (left) Atlantic, and (right) Indo-Pacific Ocean north of 34.5°S (indicated by vertical dashed lines) and globally within the Southern Ocean (“S.O.”). The zero-streamline contour is defined by the solid black line.

**Extended Data Fig. 4:**
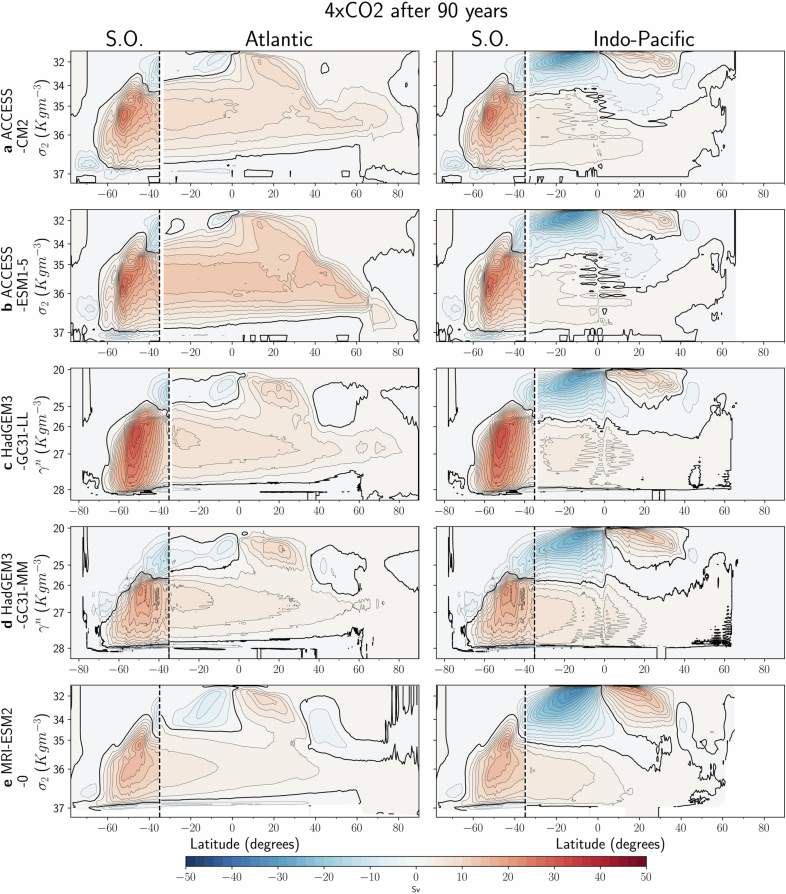
Overturning circulation in the future state of the 4xCO2 experiment in density space. Overturning streamfunction (Sverdrups (Sv); 2 Sv contour interval) in density coordinates, using neutral density (*γ*^n^) or density referenced to 2000 m (σ_2_) averaged over the 20-year period centred on year 90 of the 4xCO2 experiment in the five available models, for the (left) Atlantic, and (right) Indo-Pacific Ocean north of 34.5°S (indicated by vertical dashed lines) and globally within the Southern Ocean (“S.O.”). The zero-streamline contour is defined by the solid black line.

In the Indo-Pacific, the anticlockwise overturning circulation not only weakens^3,31^ but also rapidly reverses (Fig. 1d and Extended Data Figs. 2 and 3), indicating the presence of an Atlantic–Pacific see-saw^27^ in the CMIP6 models. The resulting clockwise PMOC appears in 91% (86%) of models in the future state of 4xCO2 (u03_hos), with variable strength (Fig. 3a,c). Its mean strength at 34.5° S is 4.9 Sv in 4xCO2 (6.4 Sv for u03_hos subset), but only 2.3 Sv in u03_hos. Similar changes in the Indo-Pacific are found in depth and density space (Extended Data Fig. 4).

**Extended Data Fig. 5:**
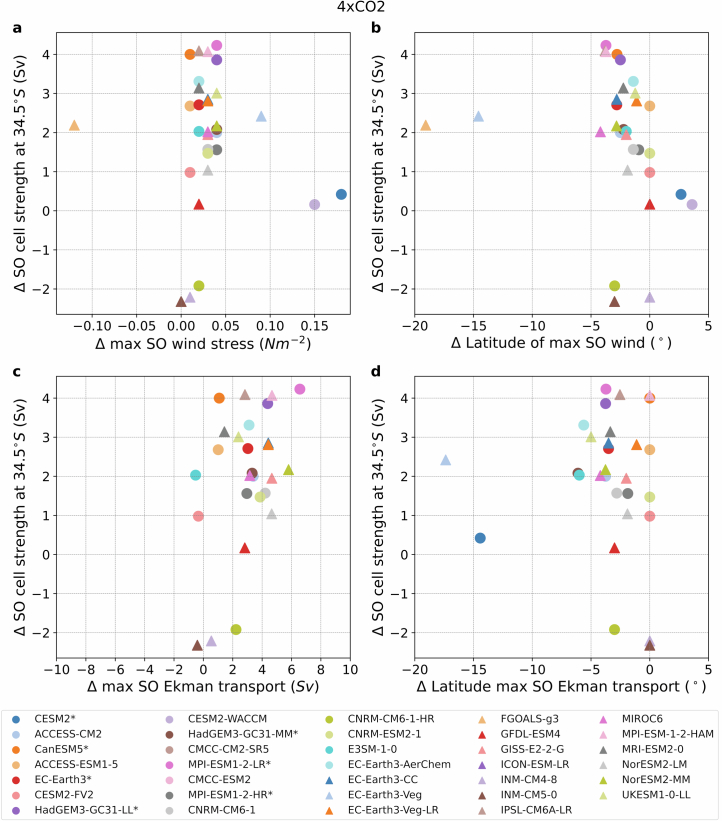
SO wind changes do not explain the intermodel spread in the SO overturning response to extreme GHG forcing. Changes in **(a)** the magnitude and **(b)** the latitude of the maximum SO wind stress, against the change in SO upper cell strength at 34.5°S (future state minus control) in the 4xCO2 experiment. **(c,d)** The same as the upper panels, but using (**c**) the magnitude and (**d**) the latitude of the maximum SO Ekman transport.

**Extended Data Fig. 6:**
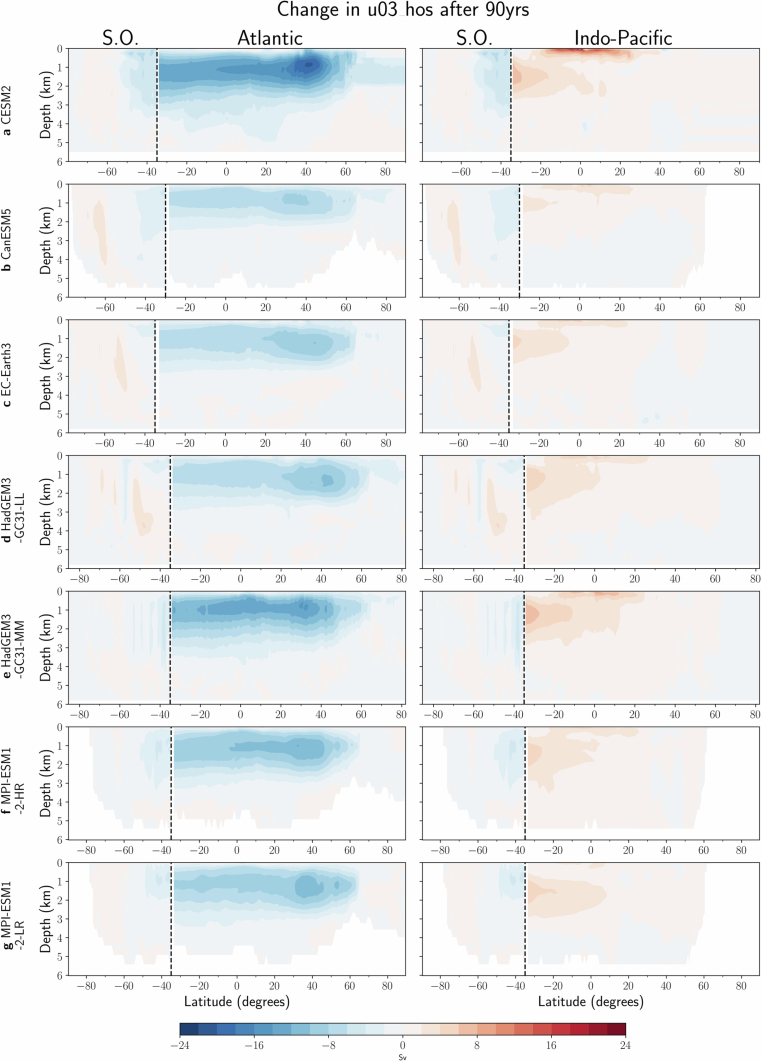
Changes in overturning circulation after 90 years of North Atlantic freshwater forcing. Differences in the overturning streamfuction change (2 Sv contour intervals) in depth space between the future state (average over the 20-year period centred on year 90) of u03_hos and the initial 50 years of the pre-industrial control experiment. The overturning circulation is plotted for the (left) Atlantic, and (right) Indo-Pacific Ocean north of 34.5°S (indicated by vertical dashed lines) and globally within the Southern Ocean (“S.O.”).

The SO upper cell at 34.5° S strengthens in 4xCO2 (Extended Data Fig. 5a) primarily owing to enhanced SO westerly winds^32,33^, which more than compensates for the weakening from polewards shifts (Extended Data Fig. 5b). Conversely, the SO upper cell weakens in u03_hos (Fig. 3d), probably owing to a polewards shift in the SO westerly winds—characterized by a dipole in SO overturning changes (Extended Data Fig. 6)—without a concurrent strengthening of the SO winds^34^.

**Extended Data Fig. 7:**
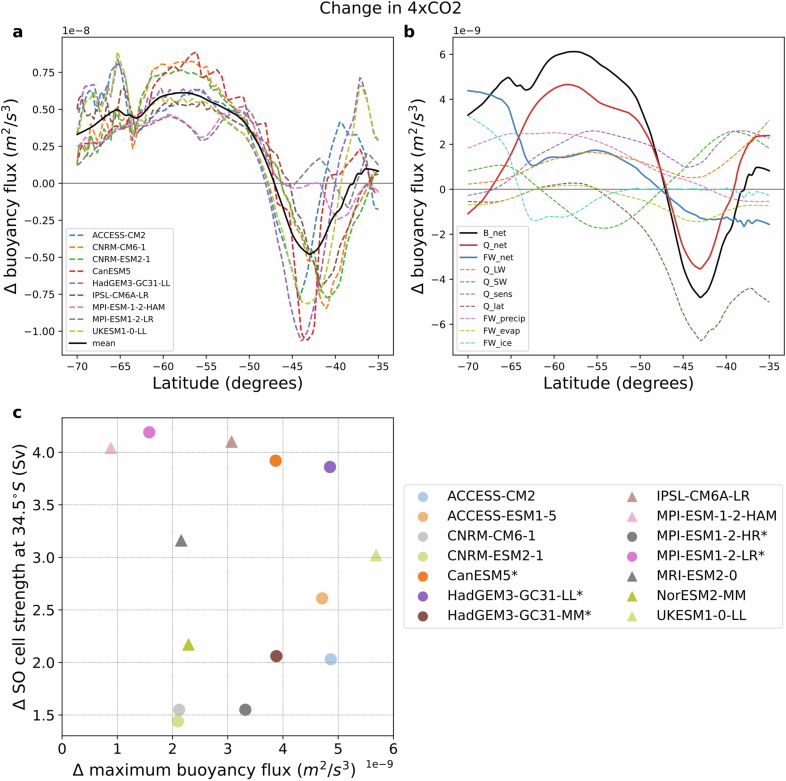
Changes in zonal-average SO surface buoyancy fluxes under 4xCO2 forcing. Changes (future state minus control) in **(a)** net surface buoyancy fluxes (B_net) across 9 CMIP6 models and **(b)** multi-model mean of surface buoyancy flux components in the 4xCO2 experiment, including net heat (Q_net) and freshwater (FW_net) fluxes and their components - longwave (Q_LW) and shortwave (Q_SW) radiation, latent (Q_lat) and sensible (Q_sens) heat fluxes, precipitation (FW_precip), evaporation (FW_evap), and sea-ice freshwater (FW_ice) fluxes. A positive change indicates a tendency towards surface buoyancy gain. (**c**) Changes in the maximum positive SO buoyancy flux against changes in SO upper cell strength at 34.5°S in the 4xCO2 experiment.

Changes in SO surface buoyancy fluxes can also strengthen the SO upper cell^21,35^. In 4xCO2, surface buoyancy gains between 70° S and 50° S, owing to reduced outwards net longwave radiation and sensible heat fluxes and increased precipitation (Extended Data Fig. 7a,b and Methods), enable the SO upper cell to shift southwards and strengthen. Conversely, surface buoyancy loss north of 50° S, owing to increased evaporation and outwards latent heat fluxes (Extended Data Fig. 7b), potentially increase the transformation and subduction of surface waters^33^. Although changes in the SO wind stress and buoyancy fluxes may be smaller under more realistic GHG forcing, they are still expected to strengthen the SO upper cell^32,33^.

As changes in maximum SO wind stress and Ekman transport in 4xCO2 do not significantly correlate (*P* > 0.05) with changes in the SO upper cell strength at 34.5° S (Extended Data Fig. 5), other processes must contribute to the intermodel spread in SO upper cell strength changes (about 4 Sv). Although intermodel differences in SO buoyancy flux changes (Extended Data Fig. 7a) may have a role^36^, there is no significant correlation (*P* > 0.05) between increases in peak SO buoyancy gain and SO upper cell strengthening (Extended Data Fig. 7c). Differences in eddy compensation, dependent on model resolution and eddy parameterization^24,25^, may also contribute to the intermodel spread. Notably, in 4xCO2, lower-resolution models from the HadGEM3, MPI, CNRM and EC-Earth3-Veg model groups have greater strengthening of the SO upper cell (Extended Data Fig. 5), implying that they have weaker eddy compensation.

## AMOC resilience under extreme forcing

**Extended Data Fig. 8:**
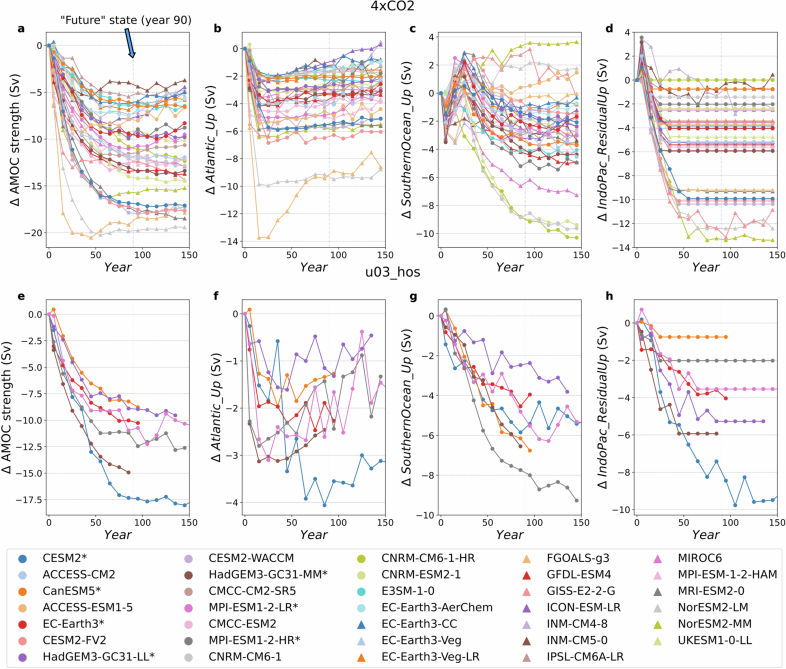
The AMOC and its upwelling pathways change under extreme forcing. Decadal-mean changes relative to the control are shown for **a-d** abrupt quadrupling of CO_2_ (“4xCO2”) and **e-h** North Atlantic freshwater hosing (“u03_hos”) forcing scenarios. Variables plotted are (**a,e**) AMOC strength, and (**b,f**) Atlantic, (**c,g**) Southern Ocean, and (**d,h**) Indo-Pacific residual upwelling pathways of the AMOC. Models used in both the 4xCO2 and u03_hos scenarios are labelled *.

**Extended Data Fig. 9:**
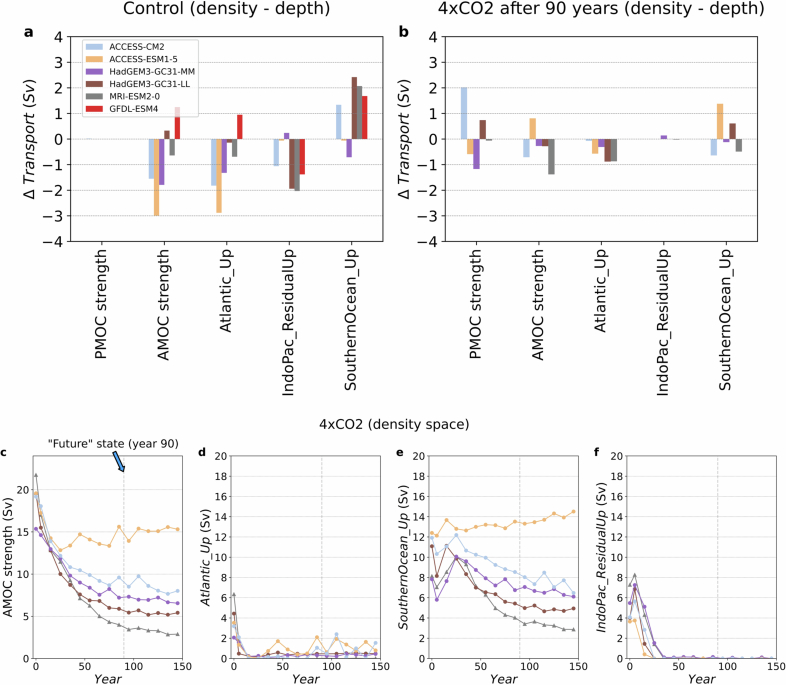
Overturning circulation strength and AMOC upwelling pathways are similar in depth and density space. **(a,b)** Differences (density space minus depth space) in these variables in **a** the control simulation and **b** the future state of the 4xCO2 experiment. (**c-f**) Decadal-mean evolution of (**c**) AMOC strength, and (**d**) Atlantic, (**e**) Southern Ocean, and (**f**) Indo-Pacific residual upwelling pathways of the AMOC in density space (Methods). The GFDL-ESM4 model is only plotted in **a**.

Under the extreme climate forcings, all AMOC upwelling pathways ultimately weaken to conserve volume as the AMOC weakens, ensuring upwelling balances downwelling, but there is large intermodel spread (Fig. 2b–d,f–h). The Atlantic (Fig. 2b,f) and Indo-Pacific residual (Fig. 2d,h) upwelling pathways weaken to approximately zero in most models after 90 years of forcing owing to AMOC shoaling, which reduces ‘cell overlap’ between the AMOC and the SO lower cell (Fig. 1a and Extended Data Figs. 2 and 3), cutting off the AMOC’s main pathway into the Indo-Pacific Ocean^37–39^ (compare Fig. 1c,d). Rapid changes in the Indo-Pacific meridional overturning circulation triggered by wave processes as the AMOC weakens^31^ also reduce the Indo-Pacific residual upwelling pathway. The future Atlantic upwelling pathway is greater in u03_hos than in 4xCO2, but it is diminished in both scenarios (<2 Sv in most models; Fig. 2b,f). In 4xCO2, the SO upwelling pathway initially increases (over years 10 to 30) owing to SO upper cell strengthening, before it decreases (Fig. 2c). The future SO upwelling pathway is therefore greater in 4xCO2 than in u03_hos (Extended Data Fig. 8c,g), but it is the dominant upwelling pathway (2–13 Sv) in both scenarios (Fig. 2c,g), sustaining the future AMOC. This suggests that without SO upwelling, the AMOC would nearly collapse under these forcings as the Atlantic and Indo-Pacific residual upwelling pathways would still diminish owing to AMOC shoaling^38^. Despite small quantitative differences in AMOC (and PMOC) strength, and in the AMOC’s upwelling pathways when calculated in density rather than depth space (Extended Data Fig. 9a,b), the AMOC’s SO upwelling pathway remains dominant in the future state (Extended Data Fig. 9c–f), affirming our findings.

**Fig. 4:**
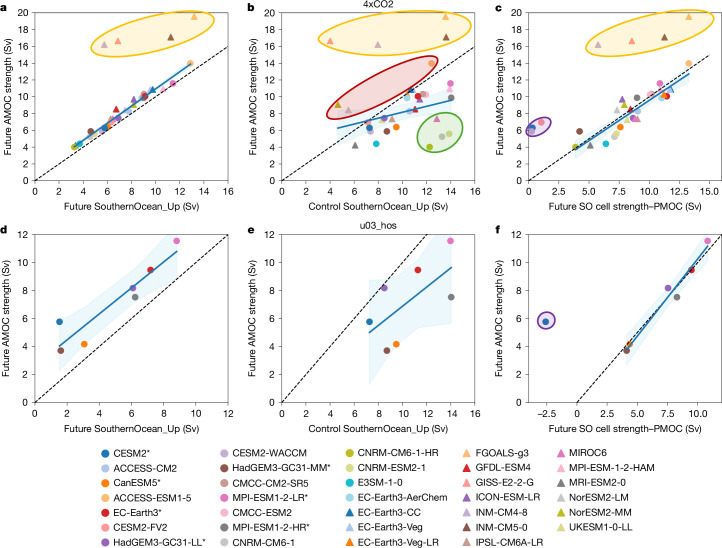
Future SO upwelling and PMOC strength determine future AMOC strength under extreme forcing. **a**–**f**, Future (**a**,**d**) and control (**b**,**e**) SO upwelling pathways of the AMOC, and combination of future SO upper cell strength at 34.5° S and inverted future PMOC strength at 34.5° S, against future AMOC strength (**c**,**f**). The future state is 90 years into the 4xCO2 (**a**–**c**) and North Atlantic freshwater hosing (u03_hos; **d**–**f**) forcing scenarios. A line of best fit across the whole ensemble, excluding outlying models shaded in orange (**a**–**c**) and purple (**c**,**f**) is shown, with blue shading indicating the 95% confidence interval. A line of equality (dashed line) is also shown. Models shaded in red and green (**b**) show relatively strong and weak future AMOC strength, respectively.

As the future SO upwelling pathway primarily sustains the future AMOC, their magnitudes are directly proportional in both 4xCO2 (*r* = 0.99; Fig. 4a) and u03_hos (*r* = 0.93; Fig. 4d), excluding 4 outlying models in 4xCO2 (shaded orange in Fig. 4a). These four models have anomalously strong future AMOCs owing to notable Atlantic or Indo-Pacific residual upwelling pathways (Fig. 2a–d). These upwelling pathways are unrealistically large in the control simulations relative to the observed AMOC strength (Fig. 2b,d) or they have anomalously weak decreases in the future state (Fig. 2b,d, brown triangles).

If SO upwelling pathway changes are consistent across models and thus the control SO upwelling pathway is directly proportional to its future magnitude, it would also correlate strongly with the future AMOC strength. Excluding the 4 outlying models, correlation between these variables is statistically significant but weak in 4xCO2 (Fig. 4b; *r* = 0.38; *P* < 0.05 (Methods)), whereas it is insignificant in u03_hos (Fig. 4e; *r* = 0.65, *P* > 0.05). Thus, although a stronger SO upwelling pathway (and SO upper cell at 34.5° S) in the control is associated with a stronger future AMOC, the relationship is weak.

## Remote ocean impacts on the future AMOC

**Fig. 5:**
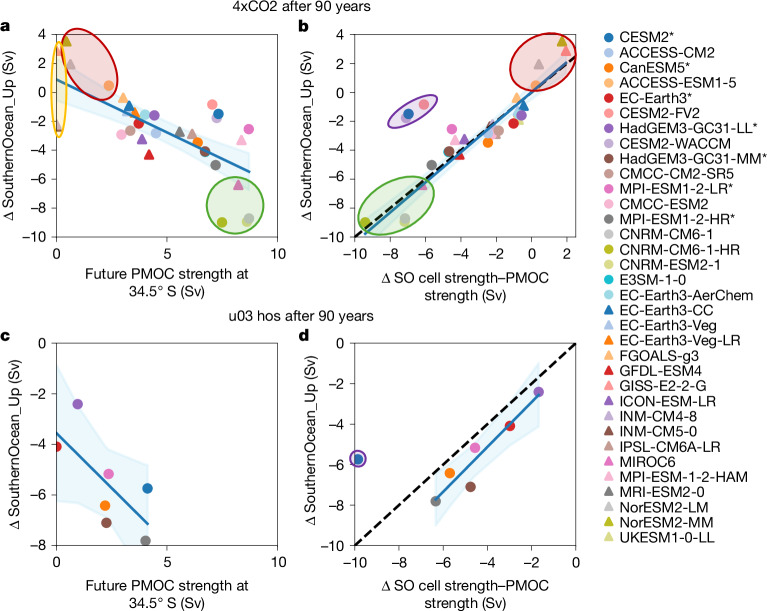
Indo-Pacific and SO overturning changes drive changes in AMOC’s SO upwelling pathway under extreme forcing. **a**–**d**, Correlation of changes in the AMOC’s SO upwelling pathway with projected future PMOC strength at 34.5° S (**a**,**c**), and the combined effect of changes in SO upper cell strength at 34.5° S and inverted future PMOC strength at 34.5° S (**b**,**d**). Changes are between the control simulation and the future state 90 years after applying 4xCO2 (**a**,**b**) or North Atlantic freshwater hosing (u03_hos; **c**,**d**) forcing scenarios. A line of best fit across the whole ensemble (**a**,**c**), excluding outlying models shaded in purple (**b**,**d**), is shown, with blue shading indicating the 95% confidence interval. A line of equality (dashed line) is shown in **b** and **d**. Models shaded in red and green (**a,b**) have enhanced or greatly weakened future Southern Ocean upwelling pathways, respectively.

In 4xCO2, the AMOC’s SO upwelling pathway weakens (Extended Data Fig. 8c), despite the strengthening of the SO upper cell at 34.5° S in 86% of models (Extended Data Fig. 5). We attribute this weakening to the PMOC that emerges in the Indo-Pacific (Fig. 3a), possibly instigated by North Atlantic forcing. The PMOC upwells via the SO upper cell, thereby reducing the volume of AMOC origin waters that can upwell in the SO, leading to a weakening of the AMOC’s SO upwelling pathway (Fig. 5a).

**Fig. 6:**
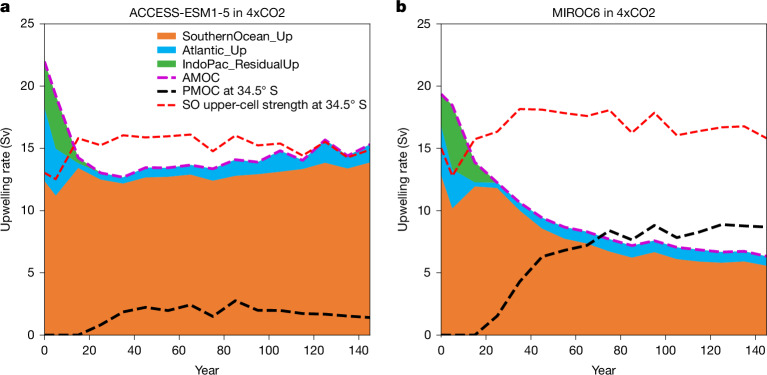
PMOC strength impacts AMOC decline under 4xCO2 forcing. **a**,**b**, Aggregated contributions of each AMOC upwelling pathway—SouthernOcean_Up (orange), Atlantic_Up (blue) and IndoPac_ResidualUp (green)—to the AMOC strength (purple line) for a model that develops a weak (**a**) and a strong (**b**) PMOC at 34.5° S (black line). The SO upper cell strength at 34.5° S (red line) is also shown.

Variability in PMOC strength across models largely explains why seven models in 4xCO2 (besides the four prementioned unrealistic models) have enhanced (shaded red) or greatly weakened (shaded green) future SO upwelling pathways (Fig. 5a). These differences result in anomalously strong or weak future AMOCs, respectively, relative to their control SO upwelling pathways (Fig. 4b). Thus, models with relatively strong future AMOCs (shaded red in Fig. 4b) have weak future PMOCs (<2.5 Sv; Fig. 5a), stabilizing the AMOC (Fig. 6a). Conversely, models with relatively weak future AMOCs (shaded green in Fig. 4b) tend to have strong future PMOCs (about 8 Sv; Fig. 5a) that enable the AMOC to weaken further (Fig. 6b). However, not all models with strong future PMOCs (>5 Sv; Fig. 5a) have anomalously weak future AMOCs relative to their control SO upwelling pathways because some models are compensated by strong increases in SO upper cell strength (Extended Data Fig. 5). Therefore, changes in the AMOC’s SO upwelling pathway depend largely on changes in both the SO upper cell strength and the future PMOC strength (Fig. 5b; *r* = 0.95). Exceptions are models with a deep northwards PMOC branch at 34.5° S that stabilizes the AMOC by enabling AMOC deep waters to join the PMOC’s northwards flow via SO zonal transports, before returning to the Atlantic through SO upwelling (Methods). This connection is small in most models (<1 Sv), except CESM2-based models (purple shading in Fig. 5b,d), owing to their anomalously deep South Pacific subtropical gyre cells (Extended Data Figs. 2a and 3a).

Unlike in 4xCO2, the SO upper cell at 34.5° S weakens in u03_hos (Fig. 3d), so the AMOC’s SO upwelling pathway weakens further, despite having a weaker future PMOC (compare mutual models in Fig. 5a,c). Hence, both a weakening SO upper cell and an emerging PMOC reduce the AMOC’s SO upwelling pathway in u03_hos (Fig. 5d).

## Future fate of the AMOC

We have shown that SO wind-driven upwelling prevents an AMOC collapse under extreme climate forcing in CMIP6 climate models. The AMOC’s future strength depends not only on the future SO upper cell strength (which depends on its present-day strength and change) but also on the future PMOC strength (if it forms) (Fig. 4c,e; *r* = 0.90 (*r* = 0.98) for 4xCO2 (u03_hos), excluding anomalous models shaded in orange and purple). Previous studies^21,40^ have highlighted the role of SO wind-driven upwelling in sustaining an AMOC in simple models, but its importance in comprehensive global climate models under extreme forcing was unexplored. GHG forcing strengthens the SO westerly winds^32^, strengthening the SO upper cell^33^. Thus, for an AMOC collapse or substantial AMOC weakening to occur, a strong PMOC that upwells in the SO (Fig. 1d) is essential because SO wind-driven upwelling must be balanced by downwelling in either the Atlantic or Indo-Pacific oceans to conserve volume^30,41^.

The emergence of a PMOC as the AMOC weakens has previously been found in global-climate-model extreme forcing experiments^27–29^, but Indo-Pacific overturning changes are generally overlooked. The consistency of PMOC formation under extreme climate forcing across CMIP6 models highlights the importance of the Atlantic–Pacific see-saw^27^ for extreme AMOC weakening. Our findings suggest that a PMOC can develop without closure of the Bering Strait, in contrast to ref. ^42^. However, North Atlantic freshwater forcing in u03_hos may reduce freshwater export from the Pacific Ocean through the Bering Strait^42^, limiting the PMOC strength. In 4xCO2, Pacific freshwater export continues, contributing to AMOC weakening^43^ and potentially enabling a stronger PMOC. The PMOC involves deep-water formation, densification (Extended Data Fig. 4) and sinking (Extended Data Figs. 2 and 3), typically occurring in the subtropical North Pacific or farther south, unlike refs. ^27–29^. As Indo-Pacific density changes enable PMOC formation, the mean state of the Indo-Pacific is probably crucial for determining future PMOC strength. PMOC formation may be driven by reduced ocean freshwater transports or atmospheric freshwater fluxes into the Indo-Pacific^44,45^. In 4xCO2, mean buoyancy fluxes into the Indo-Pacific generally increase, although they decrease in some regions south of about 15° S with net surface buoyancy loss (not shown). Although mean surface buoyancy fluxes do not clearly show negative changes, temporal variations and their interaction with density fields, which impact water mass transformation^46^, could facilitate PMOC formation. In addition, wave processes emanating from the North Atlantic can reduce Indo-Pacific upwelling^31^ and potentially instigate Indo-Pacific downwelling and PMOC formation. Despite the transient state of the overturning circulation, the PMOC strength remains similar from years 90 to 150 in 4xCO2 (Fig. 3a), with the PMOC streamfunction in density (depth) space showing a similar structure across isopycnal (isobath) layers throughout this period (not shown). In addition, the PMOC’s downwelling to depths of 2 km to 5 km (Extended Data Fig. 2) suggests that the Indo-Pacific diapycnal transports in Extended Data Fig. 4 are due to water-mass transformation, not transient isopycnal heaving. Future research is needed to explore how the aforementioned processes affect PMOC formation and AMOC decline.

An active PMOC associated with a weakened or collapsed AMOC was evident in past climates, including during the Last Glacial Maximum and its termination^45,47^ and the warm Pliocene when atmospheric CO_2_ levels were similar to present-day levels^48^. Our findings suggest that PMOC formation may have facilitated AMOC collapses in past climates by reducing the SO upwelling of AMOC deep waters. The PMOC transports heat northwards^49^, and affects ocean biogeochemistry and carbon uptake^47^, highlighting the need to assess its impact on future climate in CMIP6 models.

Although SO upwelling sustains the AMOC under the extreme forcings considered here, increased forcings may further weaken the AMOC, but only if the SO upper cell weakens or the PMOC strengthens. AMOC weakening is therefore resisted through maintenance of the SO winds and freshwater input to the Indo-Pacific Ocean, implying that a large forcing is required for an AMOC collapse^11,14^. Hence, we argue that changes in the hydrological cycle or changes in freshwater transport into the Indo-Pacific Ocean, in addition to the Atlantic Ocean^14^, may be crucial for AMOC tipping by enabling formation of a strong PMOC. Although the models show a PMOC does not develop or remains generally weak in realistic future scenarios^3^, if they underestimate the possibility of a strong PMOC forming, they may also underestimate the risk of a future AMOC collapse. Therefore, understanding model biases is essential.

Our findings highlight the need for improved observational estimates of the SO and Indo-Pacific Ocean overturning circulations and their heat and freshwater transports, similar to efforts in the South Atlantic^50^. This would allow us to identify climate models with realistic present-day SO upper cell strengths and to detect changes in the AMOC’s upwelling pathways and transports owing to global warming. Constraining the AMOC’s upwelling pathways would also improve predictions of AMOC weakening under realistic forcing scenarios^3^.

The future extent of AMOC decline remains uncertain^1,5,10,13^, despite its critical impact on heat, carbon and nutrient transport^6,7^, and thus on global climate^2^ and ecosystems. We have shown that SO wind-driven upwelling prevents an AMOC collapse in CMIP6 climate models under extreme GHG and North Atlantic freshwater forcings. With predicted stronger SO winds and the present-day Bering Strait open, we conclude that a twenty-first century AMOC collapse (defined here as weakening to below 6 Sv) is unlikely. However, to refine AMOC projections, a greater focus on ocean circulation changes beyond the North Atlantic and their driving mechanisms is essential. Future changes in both the Atlantic and Pacific overturning would impact regional weather, climate, ecosystems and agriculture^2,10^, so accurate projections of both are required to inform adaptation and resilience to climate change.

## Methods

### Models and experiments

We analyse the AMOC upwelling pathways in 34 CMIP6 models (Extended Data Table 1) from the pre-industrial control (piControl) simulation^51^ and their responses under two extreme-forcing scenarios: the ‘abrupt-4xCO2’ experiment^51^ (‘4xCO2’ herein) and the ‘u03_hos’ experiment from the North Atlantic Hosing Model Intercomparison Project^19^ (NAHosMIP). In 4xCO2, atmospheric CO_2_ concentrations are instantaneously quadrupled from piControl levels and maintained for 150 years. In u03_hos, a uniform freshwater forcing of 0.3 Sv is applied to the North Atlantic between 50° N and the Bering Strait for at least 100 years. We examine seven CMIP6 models in u03_hos. We use a single ensemble member from each run (Extended Data Table 1). All available models are included in our analysis to ensure a wide range in the AMOC’s upwelling pathways, enabling robust relationships to be inferred.

### Variables

We analyse the monthly mean overturning mass streamfunction, including both Eulerian mean and parameterized eddy components^34^, in depth space (variables, ‘msftmz’ or ‘msftyz’) and in density space (variables, ‘msftmrho’ or ‘msftyrho’), in six models that provide this variable or in which we calculate it. We average the overturning streamfunction over the first 50 years of the piControl simulation, and over the 20-year period centred on 90 years into the 4xCO2 and u03_hos experiments to obtain the ‘future’ state. We focus on the period 90 years in the extreme-forcing experiments because it is available in all models and because the AMOC has generally stabilized. We calculate the AMOC strength (where AMOC is defined as the Atlantic Ocean mid-depth overturning cell) from the maximum Atlantic streamfunction value north of the Equator and below 500 m depth. We calculate the PMOC strength from the maximum Indo-Pacific streamfunction value at 34.5° S, between 500 m and 4,000 m depth, to exclude wind-driven gyres and unrelated deep overturning circulations. Although weak PMOC cells are present in the control experiment of a few models (Fig. 3a,c), they are confined to the deep ocean and thus do not affect the magnitude of the AMOC’s upwelling pathways. In density space, the AMOC and PMOC strengths are defined similarly to those in depth space, but at a neutral density greater than 1,026 kg m^−3^ in the HadGEM3 models and at a density referenced to 2,000 m greater than 1,035 kg m^−3^ in 4 other models analysed (Extended Data Fig. 4).

### Calculating the AMOC’s upwelling pathways

#### Upwelling pathway definitions

We apply the method of ref. ^3^, adapted from ref. ^37^, to calculate the AMOC’s upwelling pathways (Fig. 1a). These pathways quantify the time-mean area-integrated volume transports that return deep waters from the AMOC’s southwards branch to its shallower northwards branch (Fig. 1c). We define the AMOC strength as its maximum strength below 500 m depth in the North Atlantic (Fig. 1a, purple circle). The upwelling pathways—Atlantic (‘Atlantic_Up’), Indo-Pacific residual (‘IndoPac_ResidualUp’) and SO (‘SouthernOcean_Up’)—define the regions where AMOC origin waters upwell before rejoining the northwards branch of the AMOC. Each upwelling pathway is greater than or equal to zero and collectively match the AMOC strength (equation (4) and Fig. 1) as the global overturning circulation conserves volume.

We determine the three upwelling pathways by analysing the zonally integrated meridional overturning streamfunction in the Atlantic and Indo-Pacific oceans, and a globally integrated streamfunction in the SO, defined as latitudes south of 34.5° S (Fig. 1). These have units of sverdrups (10^6^ m^3^ s^−1^). The Atlantic upwelling pathway (Atlantic_Up, blue box) corresponds to the upwelling rate of AMOC deep waters in the Atlantic Ocean that return northwards nearer the surface, inferred from the closed streamlines of the AMOC (Fig. 1a). The SO upwelling pathway (SouthernOcean_Up, orange box) quantifies total upwelling of North Atlantic (that is, AMOC) origin waters by the SO upper cell, including those that first upwell in the Indo-Pacific (Fig. 1). The Indo-Pacific residual upwelling pathway (IndoPac_ResidualUp, green box in Fig. 1b) quantifies the AMOC’s upwelling pathway in the Indo-Pacific Ocean that does not later upwell in the SO (the latter is accounted for by SouthernOcean_Up). Upwelling of AMOC origin waters in the South Indo-Pacific Ocean subtropical gyre cells that first upwell in the SO (most notable in CESM2-based models) are also accounted for by SouthernOcean_Up, not IndoPac_ResidualUp. We calculate the Indo-Pacific residual upwelling pathway as a residual using equation (4), which ensures volume conservation in the ocean.

In ref. ^3^, we showed that in a transient state, changes in AMOC strength are balanced by changes in the AMOC’s upwelling pathways, and vice versa. These changes are communicated rapidly by non-advective wave processes^31^, ensuring a global upwelling–downwelling balance to conserve volume. Thus, on a decadal or longer timescale, the time-mean AMOC upwelling pathways are equal to the time-mean AMOC strength (Fig. 2). Changes in the meridional overturning circulation remote from, but connected to, the North Atlantic through the overturning streamfunction therefore modulate the AMOC strength, even if the AMOC weakening is instigated by changes in North Atlantic forcing.

#### Equations

**Extended Data Fig. 10:**
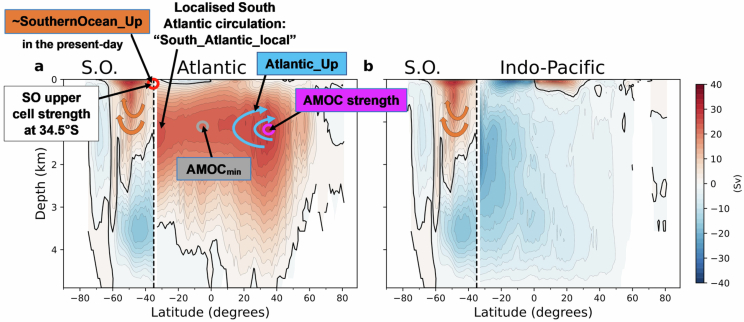
Localised South Atlantic overturning circulation can reduce SO upwelling of the AMOC. (**a,b**) Meridional overturning streamfunction (Sverdrups (Sv); 2 Sv contour interval) from the GISS-E2-2-G pre-industrial control simulation, illustrating the method used to separate the AMOC’s upwelling pathways when the AMOC has a localised South Atlantic circulation that is isolated from the North Atlantic. Streamfunctions are shown for the Atlantic (**a**) and Indo-Pacific (**b**) Oceans north of 34.5°S (indicated by the vertical dashed line) and globally within the Southern Ocean (“S.O.”). The 0-Sv streamline is marked by a solid black line. The colored circles highlight the MOC strength at the highlighted location.

The equations used to calculate the upwelling pathways (equations (1)–(4)) are:

1 $${\rm{Atlantic}}\_{\rm{Up}}={{\rm{AMOC}}}_{\max }-{{\rm{AMOC}}}_{\min }$$

2 $${\rm{South}}\_{\rm{Atlantic}}\_{\rm{local}}={\rm{AMOC}}\_34{\rm{S}}-{{\rm{AMOC}}}_{\min }$$

3 $$\begin{array}{c}{\rm{S}}{\rm{o}}{\rm{u}}{\rm{t}}{\rm{h}}{\rm{e}}{\rm{r}}{\rm{n}}{\rm{O}}{\rm{c}}{\rm{e}}{\rm{a}}{\rm{n}}{\rm{\_}}{\rm{U}}{\rm{p}}=\text{min}({\psi }_{\text{max}}{|}_{\phi =34.{5}^{^\circ }{\rm{S}}}-{\rm{S}}{\rm{o}}{\rm{u}}{\rm{t}}{\rm{h}}{\rm{\_}}{\rm{A}}{\rm{t}}{\rm{l}}{\rm{a}}{\rm{n}}{\rm{t}}{\rm{i}}{\rm{c}}{\rm{\_}}{\rm{l}}{\rm{o}}{\rm{c}}{\rm{a}}{\rm{l}}\\ \,\,\,\,\,-{\rm{P}}{\rm{M}}{\rm{O}}{\rm{C}}{|}_{{\rm{z}}{\rm{\_}}{\rm{A}}{\rm{M}}{\rm{O}}{\rm{C}}{\rm{\_}}34{\rm{S}}},{{\rm{A}}{\rm{M}}{\rm{O}}{\rm{C}}}_{\text{min}})\end{array}$$

4 $${\rm{IndoPac}}\_{\rm{ResidualUp}}={{\rm{AMOC}}}_{\max }-{\rm{Atlantic}}\_{\rm{Up}}-{\rm{SouthernOcean}}\_{\rm{Up}}$$

where AMOC_max_ is the maximum AMOC strength north of the Equator (referred to as the AMOC strength in the main text; purple circle in Fig. 1a), AMOC_min_ (≥0 Sv) is the minimum value among the maximum AMOC strengths simulated at each latitude, calculated between 34.5° S and the Equator (grey circle in Extended Data Fig. 10), AMOC_34S is the maximum AMOC strength at 34.5° S, PMOC∣_z_AMOC_34S_ is the PMOC strength (≥0 Sv) at 34.5° S at the depth of AMOC_34S, and *ψ*_max_∣_*ϕ* = 34.5°S_ is the maximum (at any depth) of the globally integrated SO upper cell strength at 34.5° S (red circle in Fig. 1a).

#### South Atlantic and Indo-Pacific overturning cells

In some models, a weak, localized South Atlantic circulation at 34.5° S, isolated from the North Atlantic, upwells in the SO (Extended Data Fig. 10). This circulation, denoted ‘South_Atlantic_local’ (equation (2) and Extended Data Fig. 10), is accounted for when calculating both the Atlantic upwelling pathway (equation (1)) and the SO upwelling pathway (equation (3)) as it reduces SO upwelling (that is, the globally integrated SO upper cell strength at 34.5° S, *ψ*_max_∣_*ϕ* = 34.5°S_ (red circle in Fig. 1a)) available for upwelling AMOC deep waters. ‘South_Atlantic_local’, determined from equation (2), is reduced if a component of the localized South Atlantic waters enters an anticlockwise overturning cell in the Indo-Pacific Ocean, upwells and rejoins the northwards branch of the localized South Atlantic circulation. This is because these waters do not upwell in the SO and thus do not reduce SouthernOcean_Up.

Under extreme forcing, most models develop a PMOC that upwells in the SO, further reducing the amount of SO upwelling available to upwell AMOC deep waters. We therefore modify the method of ref. ^3^ to account for the presence of a PMOC. A latitudinally expansive PMOC at the depth of the AMOC maximum at 34.5° S, z_AMOC_34S, indicates that any AMOC waters that enter and upwell in the Indo-Pacific Ocean must later upwell in the SO to rejoin the AMOC’s northwards branch, as they cannot bypass the PMOC. This scenario occurs in the future state of all models with a PMOC (Extended Data Figs. 2 and 3), ensuring that IndoPac_ResidualUp is zero (Fig. 2d,h). If SouthernOcean_Up, calculated from the first expression on the right-hand side of equation (3), exceeds the AMOC transport into the SO (AMOC_min_; grey circle in Extended Data Fig. 10), we adjust SouthernOcean_Up to match AMOC_min_ (second expression on the right-hand side of equation (3)). This ensures that the AMOC’s SO upwelling pathway is not stronger than the AMOC transport into the SO, as required by conservation of volume. We therefore implicitly account for the impact of the PMOC on the AMOC’s SO upwelling pathway in this case, based on conservation of volume. We account for the PMOC implicitly rather than explicitly to prevent inaccuracies in the upwelling pathways that would otherwise occur in the following scenarios.

1. If the PMOC maximum is below z_AMOC_34S, then AMOC deep waters can enter the PMOC’s northwards branch before upwelling in the SO (most notable in CESM2-based models).
2. If a localized clockwise overturning cell at 34.5° S is present in the upper Pacific Ocean (found in the control state of a few models; for example, Extended Data Fig. 1f,g), then SO upper cell waters enter the northwards near-surface branch of these Pacific cells, sink and later enter the AMOC’s northwards branch via SO zonal transports.

In these scenarios, not all the southwards PMOC transport at 34.5° S reduces the AMOC’s SO upwelling pathway (SouthernOcean_Up), so explicitly accounting for the maximum PMOC strength at 34.5° S would underestimate this upwelling pathway.

We therefore only explicitly account for upwelling of PMOC deep waters in the SO if they cannot be connected to the AMOC (first expression on the right-hand side of equation (3)), unlike in the scenarios above. Thus, we subtract the PMOC strength at the depth of the maximum AMOC strength at 34.5° S, z_AMOC_34S, from *ψ*_max_∣_*ϕ* = 34.5°S_ when calculating SouthernOcean_Up (equation (3)). Hence, if the PMOC maximum is shallower (deeper) than z_AMOC_34S, we subtract the magnitude of the southwards (northwards) PMOC transport below (above) z_AMOC_34S to calculate SouthernOcean_Up. This results in a small decrease in SouthernOcean_Up during the initial 30 years of the forcing experiments relative to not explicitly accounting for these PMOC transports.

Our approach is validated by IndoPac_ResidualUp tending rapidly (over several decades) towards zero (Fig. 2d,h) before we constrain it to zero by setting SouthernOcean_Up to AMOC_min_ (second expression on the right-hand side of equation (3)). The PMOC rapidly expands, preventing upwelling above the PMOC’s base. Therefore, IndoPac_ResidualUp quickly tends to zero when a PMOC forms. Our approach is further validated by the strong correlation between the inverse future PMOC strength and the change in the AMOC’s SO upwelling pathway (Fig. 4a,c), despite our approach not explicitly accounting for the PMOC in the future-state calculations (when SouthernOcean_Up is set to AMOC_min_). The outlying CESM2-based models in Fig. 4c,f and Fig. 5b,d (purple shading) emphasize the importance of implicitly accounting for the PMOC in the future state to prevent inaccuracies in the magnitude of SouthernOcean_Up (see scenario 1 above). We further validated our method by examining the overturning streamfunctions across models and experiments for inconsistencies with their calculated upwelling pathways.

#### Southern Ocean buoyancy fluxes

Following ref. ^52^, we calculate the surface buoyancy flux, *B*, across 16 CMIP6 models using equation (5):

5 $$B=\left(\frac{g}{{\rho }_{0}}\right)\,\left[\frac{\alpha {Q}_{{\rm{H}}}}{{c}_{p}}+\beta {S}_{0}{Q}_{{\rm{F}}}\right]$$

where *g* is the acceleration due to gravity, *ρ*_0_ is the reference density of seawater and *S*_0_ is the reference salinity. The net surface heat (*Q*_H_; W m^−2^) and freshwater (*Q*_F_; kg m^−2^ s^−1^) fluxes are positive for surface ocean inputs of heat and freshwater, respectively. We allow the thermal expansion and saline contraction coefficients (*α* and *β*) to vary with latitude and time, using the sea surface temperature and salinity variables (‘tos’ and ‘sos’, respectively) from each model. The net surface heat and freshwater flux variables (‘hfds’ and ‘wfo’, respectively) include contributions from sea-ice fluxes. We separate the heat and freshwater fluxes into their components—the net longwave and shortwave radiation (‘rls’ and ‘rss’), latent and sensible heat fluxes (‘hfls’ and ‘hfss’), precipitation and evaporation fluxes (‘pr’ and ‘evspsbl’), and sea-ice freshwater fluxes that are calculated as a residual. We calculate the multi-model mean components from a subset of nine models that provide all these variables (Extended Data Fig. 7b). We find that using a time-varying thermal expansion coefficient causes large shifts in the changes in the heat flux components, but these shifts mostly compensate each other, resulting in only minor differences in the net surface heat and buoyancy flux changes (a slight reduction in the maximum positive changes). Therefore, we show changes in the heat flux components using the thermal expansion coefficient from the control simulation (Extended Data Fig. 7b) to accurately determine the cause of the net buoyancy flux changes.

### Significance tests

We conduct a two-tailed Student’s *t*-test to assess significance of correlations between variables. A *P* value below 0.05 is considered significant, indicating a 95% confidence level.

## Online content

Any methods, additional references, Nature Portfolio reporting summaries, source data, extended data, supplementary information, acknowledgements, peer review information; details of author contributions and competing interests; and statements of data and code availability are available at 10.1038/s41586-024-08544-0.

## Figures and Tables

**Extended Data Table 1:**
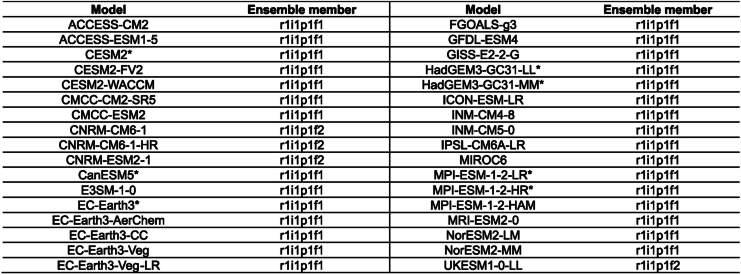
Climate models and their ensemble members

The 34 CMIP6 models from the pre-industrial control and abrupt-4xCO2 experiments used in this study (Methods). Models with * are also used in the u03_hos experiment^53^.

## Data Availability

The pre-industrial control and 4xCO2 experiment CMIP6 data used in this study are available at https://esgf-index1.ceda.ac.uk/search/cmip6-ceda/. The NAHosMIP u03_hos experiment data^53^ used in this study are available on Zenodo at 10.5281/zenodo.7643437 (ref. ^54^).

## Code availability

The code used to calculate the AMOC’s upwelling pathways is available at 10.5281/zenodo.11116004 (ref. ^55^).
