# Author Correction: Continued Atlantic overturning circulation even under climate extremes

**DOI:** 10.1038/s41586-025-08977-1

**Published:** 2025-04-17

**Authors:** J. A. Baker, M. J. Bell, L. C. Jackson, G. K. Vallis, A. J. Watson, R. A. Wood

**Affiliations:** 1 Met Office, Exeter, UK; 2 University of Exeter, Exeter, UK

**Keywords:** Physical oceanography, Projection and prediction, Physical oceanography, Climate and Earth system modelling

Correction to: *Nature* 10.1038/s41586-024-08544-0 Published online 26 February 2025

In the version of the article initially published, in the fourth paragraph of the “Future fate of the AMOC” section, the sentence “Although the models show a PMOC does not develop in realistic future scenarios^3^, if they underestimate the possibility of a PMOC forming…” should have read “Although the models show a PMOC does not develop or remains generally weak in realistic future scenarios^3^, if they underestimate the possibility of a strong PMOC forming…”. Additionally, in the sixth paragraph of this section, the text in parentheses has been added for clarity to the sentence “With predicted stronger SO winds and the present-day Bering Strait open, we conclude that a twenty-first century AMOC collapse (defined here as weakening to below 6 Sv) is unlikely.” Due to an error during figure preparation following peer review, the marker colours in Extended Data Fig. 5 did not correspond to the legend colour scheme. The change does not affect the results or conclusions of the study. These corrections have been made to the HTML and PDF versions of the article.

